# The Trans-Pacific Partnership Agreement and health: few gains, some losses, many risks

**DOI:** 10.1186/s12992-016-0166-8

**Published:** 2016-06-06

**Authors:** Ronald Labonté, Ashley Schram, Arne Ruckert

**Affiliations:** Faculty of Medicine, School of Epidemiology, Public Health and Preventive Medicine, University of Ottawa, 850 Peter Morand Crescent, Ottawa, K1G 3Z7 ON Canada

**Keywords:** Health policy, Social determinants of health, Trade and investment policy, Population health

## Abstract

**Background:**

In early October 2015, 12 nations signed the Trans-Pacific Partnership Agreement (TPPA), promoted as a model ‘21^st^ century’ trade and investment agreement that other countries would eventually join. There are growing concerns amongst the public health community about the potential health implications of such WTO+ trade and investment agreements, but little existing knowledge on their potential health impacts.

**Methods and results:**

We conducted a health impact review which allows for a summary estimation of the most significant health impacts of a set of policies, in our case the TPPA. Our analysis shows that there are a number of potentially serious health risks, with the following key pathways linking trade to health: access to medicines, reduced regulatory space, investor-state dispute settlement (ISDS), and environmental protection and labor rights. We also note that economic gains that could translate into health benefits will likely be inequitably distributed.

**Conclusion:**

Our analysis demonstrates the need for the public health community to be knowledgeable about trade issues and more engaged in trade negotiations. In the context of the COP21 climate change Agreement, and the UN Sustainable Development Goals, this may be an opportune time for TPPA countries to reject it as drafted, and rethink what should be the purpose of such agreements in light of (still) escalating global wealth inequalities and fragile environmental resources—the two most foundational elements to global health equity.

## Background

In early October 2015, 12 nations signed the Trans-Pacific Partnership Agreement (TPPA) (Table 1). The TPPA was promoted as a model ‘21^st^ century’ trade and investment agreement that other countries would eventually join, bypassing stalled multilateral negotiations within the World Trade Organization (WTO). There is growing interest in academia about the links between trade and health, and previous analyses have established both positive and negative impacts on health. On the positive side, if trade stimulates economic growth and economic gains trickle down to the wider population, growth in trade can contribute to improvement of health outcomes [1], as well as engendering access to novel health technologies and facilitating regulatory convergence [2]. However, there is growing concern that the new generation of 21^st^ century trade agreements is more likely to undermine health results due to their inclusion of a range of novel trade, and especially investment provisions [3].

**Table 1:** TPPA member country data

Country	GDP per capita^a^	% of total TPPA GDP	# of FTAS with TPPA members	Health expenditure (% GDP)^b^	Under 5 mortality (PER 1000)^d^
Australia	$61,925	5.19 %	7	9.4 %	4
Brunei	$40,979	0.06 %	7	2.5 %	10
Canada	$50,235	6.37 %	4	10.9 %	5
Chile	$14,528	0.92 %	10	7.7 %	8
Japan	$36,194	16.42 %	7	10.3 %	3
Malaysia	$11,307	4.62 %	7	4.0 %	7
Mexico	$10,325	1.21 %	5	6.2 %	13
New Zealand	$37,896^c^	0.59 %	6	9.7 %	6
Peru	$6603	0.72 %	6	5.3 %	17
Singapore	$55,979	1.10 %	9	4.6 %	3
United States	$52,980	62.14 %	6	17.1 %	7
Vietnam	$1908	0.66 %	6	6.0 %	22

^a^Current US$ in 2014 ^b^2013 data ^c^2011 data ^d^2015 data

Based on analyses of recent free trade agreements (FTAs) involving the U.S. (the real force behind the TPPA), and leaked draft texts of the negotiations, early public health concerns centered on potential TPPA impacts on costs to medicines, tobacco and alcohol control, diet-related regulations and public health policy-making more generally [4–8]. In November 2015 the final Agreement was released publicly. Given its 6000+ pages and that many of its provisions will be subject to interpretations by dispute or arbitration panels, the assessment that follows is preliminary. Our findings are based on a health impact assessment that covers the TPPA’s major health-related aspects. Our analysis followed a standard health impact assessment (HIA) protocol: during the screening stage we determined the need for a HIA and established various links between FTAs and health based on a review of existing frameworks in the public health literature; [4, 9–11] during the scoping stage we decided to focus predominantly on the health risks of the TPPA to keep the HIA within scope for this article; during the appraisal stage we assessed (initially leaked and later publicly released) chapters and provisions identified during the screening stage for their specific health impacts.

### Access to medicines

From the outset of negotiations it was expected that the TPPA would strengthen patent protection measures beyond those in the WTO TRIPS Agreement (that is, creating ‘TRIPS+’ obligations), and it has. The TPPA allows patenting of existing pharmaceuticals already under patent for ‘new uses, new methods of using…or new processes’. These are all TRIPS+ provisions which could increase the ‘evergreening’ of patents (the continuous issuance of new patents on essentially the same drug) beyond their original 20 years of patent protection provided by the WTO’s TRIPS Agreement. This will delay generic competition and the lowering of costs for such drugs. The TPPA also allows for an unspecified period of ‘patent term extensions’ for ‘unreasonable delays’ (more than 5 years after applying for a patent) before the drug is approved for the market. The WTO TRIPS Agreement never included this on the basis that 20 years of patent protection was adequate to account for such delays.

The most contentious provision is the inclusion of biologics for the first time in a trade agreement. Expensive to research and produce, and important for the treatment of cancer and immune disorders, the cost to individuals or insurers for these new treatments can reach hundreds of thousands of dollars annually [12, 13]. Pharmaceutical companies in the US wanted 12 years of monopoly, primarily by preventing governments from making available to generic companies the clinical trial data the companies filed when applying for their patent. This ‘data exclusivity’ provision would delay the creation of generic versions of biologic drugs, or ‘biosimilars’ (i.e. drugs made from living organisms). The TPPA provides for 8 years of such data protection, but allows Parties (the countries that ratify the Agreement) to offer only 5 years if other forms of patent protection guarantee the same 8 year minimum of market exclusivity. These provisions will slow affordable access to such products. Four TPPA countries (Mexico, Peru, Malaysia and Vietnam) are given longer compliance periods for all of the provisions on pharmaceutical intellectual property rights, but even with these extensions they are unlikely to catch up economically with the wealthier Parties’ abilities to afford costly new drugs.

The TPPA at the same time affirms that “a Party may take measures to protect public health in accordance with…the Declaration on TRIPS and Public Health’ (art.18.50, ¶3), a reference to the 2001 Doha Declaration clarifying that countries are able to issue compulsory licenses for generic drugs in a public health emergency. Although this may provide some flexibility for governments, the Doha Declaration has only rarely been invoked for conventional drugs (in the earlier 2000s, mostly by upper-middle-income countries and primarily for antiretrovirals), and one can anticipate political pressures from the US to prevent its use for biosimilars even after the period of data and market protection have passed, if the patent has not yet expired [14]. Public health lobbying and country-specific concerns also succeeded in excluding from the TPPA’s provisions pharmaceutical products and medical devices purchased under a national government procurement program (where governments negotiate for bulk purchases to get the best price) and post-market subsidisation. Reached late in the negotiations, and not what the US-based pharmaceutical industry wanted, this exclusion limits the ability of industry to affect the functioning (and drug costs) of these national programs, currently affecting just 4 countries: New Zealand, Australia, Japan and the U.S.

Since intellectual property is considered a form of investment in the TPPA, regulatory reforms to a Party’s policies could be liable to an investor suit under Investor State Dispute Settlement (ISDS) provisions (discussed later). One such case, under the North American Free Trade Agreement’s) Chapter on ISDS, concerns Eli Lilly’s USD 500 million claim against the decision by Canada, supported by its courts, to revoke patents on two drugs that failed a ‘promise doctrine’ of demonstrable utility made at the time of patent filing. This case is complex [15] and still unresolved; but it underscores the potential for ISDS to be used to challenge domestic pharmaceutical policies.

In sum, the TPPA provisions, *prima facie,* appear incoherent with the UN decision in December 2015 to establish a High-Level Panel on Access to Medicines. This panel is charged with seeking solutions to the present high cost of new medicines that prevent access for most by balancing ‘the rights of inventors, international human rights law, trade rules, and public health in the context of health technologies’ [16]. Margaret Chan recently challenged think tanks to answer crucial questions for agreements like the TPPA, including what constitutes fair profits for the pharmaceutical industry, whether market exclusivity conferred by patent protection actually stimulates innovation, and if these agreements do reduce access to medicine, can we really consider them progress at all? [17].

### Reduced regulatory space

Another public health concern is that provisions in the TPPA would likely reduce governments’ regulatory space. Three Chapters in particular do so: on Sanitary and Phytosanitary Measures (SPS), on Technical Barriers to Trade (TBT), and on Regulatory Coherence. The first two are based on similarly named agreements under the WTO system, but are ‘WTO+’ with new provisions; the third is a novel TPPA addition.

The WTO SPS Agreement defers to the *Codex Alimentarius Commission* (a body jointly administered by the WHO and the FAO) with the presumption that if a WTO member country’s food safety regulations are coherent with those in the *Codex*, there is no conflict with liberalisation obligations. The TPPA’s SPS Chapter does not directly defer to *Codex,* but rather to an unnamed group of ‘international standards, guidelines and recommendations,’ introducing potential ambiguity over what should be the referent standards. Moreover, if countries exceed whatever international standards are used as referents, they will be required to produce ‘documented and objective scientific evidence’ justifying such provisions, weakening a government’s ability to exercise the ‘precautionary principle’ when there is little evidence or scientific consensus.

Similarly, the TPPA TBT tightens WTO TBT requirements. The WTO TBT requires that governments’ technical regulations must ‘not be more trade-restrictive than necessary to fulfil a legitimate objective’. This applies to health regulations (art.2.2), creating a problematic ‘necessity test’ that has often been a basis for disputes over health and environmental regulation, wherein governments have to justify to complainant countries why the regulation is necessary to achieve a legitimate policy goal [18]. If they fail to do so, the complainant countries can initiate a trade dispute. The TPPA TBT in addition to this requirement states that “nothing in this Chapter shall prevent a Party from adopting or maintaining technical regulations or standards, *in accordance with its rights and obligations under this Agreement*” (art.8.3, ¶5, our emphasis). The highlighted text means that governments can really only regulate according to the rules under the TPPA’s TBT. One of these new TPPA rules requires Parties to ‘cooperate with each other’ in setting new international standards (the sort that *Codex* might adopt) that ‘do not create unnecessary obstacles to international trade’ (art.8.5 ¶). This may pre-empt a future TPPA dispute over such standards, but at the risk of lowering standards in deference to trade.

Both the TPPA TBT and Regulatory Coherence Chapters add new requirements for participation in regulation setting in any one TPPA country by government or ‘interested persons’ from other Parties, defined as ‘a national or an enterprise’ (art.1.3). The appeal of creating uniform regulations is understandable insofar as it reduces transaction costs for commercial firms, even as it increases the public costs of complying with the administrative and consultative requirements. More critically, these provisions open the door for ‘regulatory capture,’ [19] where transnational tobacco, alcohol, and food corporations from any TPPA country are allowed to be present in the formulation of regulations that affect their own business practices in any other TPPA country.

### Still problematic ISDS measures

The TPPA excludes the Regulatory Coherence Chapter, but not the SPS or TBT Chapters, from its ISDS (investor-state dispute settlement) provisions. ISDS is one of the most controversial inclusions in FTAs. Investment treaties are not new; over 3200 bilateral or regional treaties have been crafted since 1959, of which over 2500 remain in force [20]. Until recently, these treaties have been simple agreements intended primarily to avoid foreign investors having their assets directly expropriated, especially in countries with weak or politically compromised judicial systems. Newer treaties are more extensive, and the use of ISDS by foreign investors to sue governments over regulatory decisions that they believe have compromised the value of their investments has risen dramatically in the past decade [21]. A 2013 review of ISDS claims found that 40 cases involved health or environmental protection [22], including food safety, pharmaceuticals and tobacco control measures. Most of the environmental disputes have important indirect health implications.

There are several criticisms of how ISDS presently functions [23]:

- decisions made in closed hearings by a three-member tribunal of lawyers, some of whom have had connections with the multinational corporations whose cases they were arbitrating;
- a small ‘elite 15’ of these lawyers deciding on the majority of cases involving large awards;
- the increasing size of awards;
- the lack of any appeal process; and
- the public cost to governments of defending such a suit (averaging USD 8 million but often much higher), even if the investor loses (although their ‘win’ rates have been rising)

Government websites argue that the TPPA’s ISDS Chapter has addressed many of these concerns. It has not. It retains the requirement for a ‘minimum standard of treatment’ of foreign investments, including ‘fair and equitable treatment’ (art.9.6, ¶1), shorthanded as FET. Over the past decade, FET has become the most common standard on which investors initiate, and tribunals rule on, a dispute. FET is subject to interpretation during disputes, and the expansive interpretation of FET by tribunals is allowing investors to challenge a range of government policies, including those involving environmental and human health. The TPPA states that ‘an investor’s expectations’ by itself (the conditions under which an investor could have legitimately expected their investment to operate and which contributed to their decision to invest) is insufficient to sue for loss or damage (art.9.5, ¶4), and is claimed as a safeguard to prevent excess awards. But these expectations can nonetheless be used by a tribunal in conjunction with other arguments in their ruling, which still allows for substantial interpretative room for tribunal members.

Similar claims that the Chapter ensures the rights of Parties to regulate in the public interest are based on Article 9.15, which states that “nothing in this Chapter shall be construed to prevent a Party from adopting, maintaining or enforcing any measure *otherwise consistent with this Chapter* that it considers appropriate to ensure that investment activity in its territory is undertaken in a manner sensitive to environmental, health or other regulatory objectives” (our emphasis). However, the five italicized words effectively undermine the entire Article, since governments can undertake such regulations *only* if they abide by all the rules of the ISDS Chapter. This offers scant protection from investor suits over changes in health or environmental regulations or policy. Curiously, ‘non-discriminatory regulations^1^…designed for legitimate public welfare objectives’, including health and the environment, ‘do not constitute indirect expropriation, except in rare circumstances’ (Annex 9-B, ¶3(b)). This appears to afford more regulatory latitude in cases based on indirect expropriation (the loss of an investment’s value ‘equivalent to direct expropriation’), although it still leaves the interpretation of what constitutes a ‘legitimate’ objective or a ‘rare’ circumstance to the judgement of three investment lawyers.

The TPPA does create new avenues for greater transparency, including a provision for *amicus curiae* (‘friend of the court’) submissions from third parties, which could include public or environmental health professionals or civil society groups introducing new evidence or argument. However, there is nothing binding on a tribunal to allow such submissions or to take these submissions into account in their rulings. More importantly, there is nothing in the Chapter that corrects the lack of an appeal process or tribunalists’ conflicts-of-interest. TPPA Parties have committed to preparing a ‘Code of Conduct’ for tribunalists. As this is still to be drafted its content is unknown, but it may adopt elements of the November 12, 2015 proposed text on ISDS released by the European Commission (EC) [24]. The Code of Conduct proposed by the EC, however, is little more than exhortations to good behaviour by tribunalists, unlikely in itself to make much difference.

### Tobacco exclusion?

Public health lobbying and early support from a few TPPA countries (notably Malaysia) led to a provision allowing Parties at any time to exclude any tobacco control measure from the Agreement’s ISDS provisions. This exclusion will prevent tobacco transnationals from challenging a country’s control measures as they have done, under other existing ISDS treaties, with Australia’s plain packaging laws and Uruguay’s tobacco package warning labels. Philip Morris Asia’s case against Australia’s plain packaging laws was recently dismissed, a victory for public health, although the case was dismissed on jurisdiction, not on the merits of the case, leaving the fate of similar cases unknown [25]. Moreover, since the TPPA states that Parties are free to access any other agreement or treaty that offers greater liberalization or protection, tobacco transnationals in one TPPA country will still be able to treaty-shop and initiate ISDS suits against another TPPA country, even if both countries have accepted the exclusion. The TPPA tobacco exclusion may exert normative pressures for tribunals established under other ISDS treaties to dismiss them as frivolous, but that will have to be seen.

That TPPA Parties thought it sufficiently important to exclude tobacco control measures from ISDS, as well as taxation measures unless one of the Parties also agrees with an investor going forward in a dispute, suggests governments’ discomfort with how ISDS presently functions, at least in certain policy realms. That an exclusion was not similarly extended to public health regulations following WHO or international ‘best practices’ guidelines around food safety or security, or prevention of alcohol abuse, is disappointing even if understandable, given tobacco’s now historic ‘exceptionalism’.

### Limited environment protection and token labour rights

The TPPA follows the US’s recent practice of including Chapters on labour and environment in its FTAs, partly to contain criticism from unions and environmental groups. The TPPA Environment Chapter does contain one important gain: binding language that prohibits Parties from providing fishing subsidies that ‘affect fish stocks that are in an overfished condition’ (art.20.16, ¶5 (a)). This is a novel provision with indirect health benefits, particularly for developing countries reliant for an affordable food source on local fisheries that may be under threat from offshore ‘factory fishing’. The rest of the Chapter, however, is replete with hortatory language (such as ‘strive to ensure’ a Parties’ own laws offer ‘high levels of environmental protection’), although Parties ‘shall not’ (a forceful term) fail to enforce their own laws if doing so ‘affects trade or investment between Parties’ (art.20.3, ¶¶3,4). In other words, weak enforcement of weak environmental laws should not be used to give a TPPA Party a trade or investment advantage, which is the main concern of the Chapter and not environmental protection per se*.* Of seven multilateral environmental agreements (MEAs) generally found in recent US FTAs, the TPPA only references three, disappointing many environmental groups.

The Chapter is also essentially silent on the major environmental health issue of the day: climate change. There is one Article (20.15) encouraging cooperation in ‘transitions to a low emissions and resilient economy.’ Yet it is significant that the TPP could make subsidies that overfish limited stocks subject to a trade dispute but not do the same for fossil fuel subsidies, or to specifically exclude subsidies for non-fossil fuel ‘green energy’ alternatives from either an ISDS or state-to-state dispute.

The Labour Chapter requires Parties to ‘adopt and maintain’ labour rights as laid out in the ILO (International Labour Organization) Declaration, regarding freedom of association, collective bargaining, elimination of forced labour, abolition of child labour, and elimination of employment discrimination (art.19.3, ¶1). Commitments appear to extend only to the declaration and not the actual conventions, as the TPPA countries, with the exception of Peru and Chile, have failed to ratify the eight fundamental ILO conventions; Brunei and the US, two other TPPA Parties, have each ratified only two [26]. Moreover, a violation of the Chapter’s obligations under the ILO Declaration applies only insofar that it affects ‘trade or investment between the Parties’. Otherwise, TPPA countries can maintain whatever non-compliant labour rights practices they choose, including child labour, which is discouraged but not explicitly banned (art.19.6).

### Who benefits?

The material above forces the question: who actually benefits from such trade and investment deals? The TPPA signing was announced by governments with the standard claim of its potentially huge economic benefits. To the extent that the Agreement’s economic gains benefit all countries, are substantial, and ‘trickle down’ in a somewhat equitable fashion to all workers, there is a potentially powerful and positive health benefit as people accumulate more of the resources needed to lead a healthy life. But these outcomes are far from certain, or even likely. The most widely cited estimate of TPPA annual income gains (achieved only by 2025) average only 0.5 % of GDP across the 12 Parties, just 0.2 % more than global economic income gains (the background trend) over the same period [27]. High-income TPPA Parties will gain less, lower-income TPPA Parties more. The econometric models used to make this prediction, however, are based on full employment—that all labour loss in non-competitive sectors is absorbed by growth in competitive ones. Empirically, this has rarely been the case, which governments appear to accept given, for example, Canada’s commitment of over CAD 5 billion to two sectors (automotive and dairy farming) expected to lose as a result of the TPPA [28].

Another problem with most static models is that they assume invariant income distribution and thus do not recognize inequitable impacts of FTAs. Alternative, more dynamic econometric models come to different conclusions. A recent study using the United Nations Global Policy Model database predicts mild economic losses for developed TPPA economies (-0.04 % average annual GDP change) and insignificant growth for developing economies (+0.22 % average annual GDP change), and expects the loss of a total of 650,000 jobs across all TPPA countries. It also notes that inequality is likely going to increase further as the share of GDP going to capital will rise while the share going to labour will decline, continuing a trend that set in with the rise of neoliberalism in the 1970s [29]. Another study also expects the economic gains of the TPPA to be distributed unevenly amongst the population, with the bottom 90 % of wage earners losing ground, while the top 1 % will gain economically, even if not by much [30].

Although TPPA developing countries may do better than their wealthier trading Parties, TPPA rules that limit or foreclose the use of government procurement or state-owned enterprises to discriminate in favour of local employers, or which forbid imposing any domestic performance requirements on foreign investment, will leave national economic development policy increasingly in the hands of private companies and international investors.

## Conclusion

Given the paltry economic gains from the TPPA, and the various direct and indirect health risks it poses, from a strictly public health vantage, this is not a good Agreement. Whether it ever manages to reach ratification is an open question. Without US ratification, which may await the next Presidential election in November 2016, there is no deal. Much effort has gone into TPPA negotiations, but without a transparent or evidence-based set of arguments for its necessity or its far-reaching public policy impacts. In the context of the COP21 climate change Agreement, and the UN Sustainable Development Goals, this may be an opportune time for TPPA countries to reject it as drafted, and rethink what should be the purpose of such agreements in light of (still) escalating global wealth inequalities and fragile environmental resources—the two most foundational elements to global health equity.

## Abbreviations

CAD, Canadian Dollars; COP21, 21^st^ Conference of Parties to the United Nations Framework Convention on Climate Change; FAO, Food and Agricultural Organization; FET, Fair and Equitable Treatment; FTA, Free Trade Agreement; GDP, Gross Domestic Product; HIA, Health Impact Assessment; ISDS, Investor-State Dispute Settlement; TBT, Technical Barriers to Trade; TPPA, Trans-Pacific Partnership Agreement; TRIPs, Trade –Related Aspects of Intellectual Property Rights; SPS, Sanitary and Phytosanitary Measures; UN, United Nations; WHO, World Health Organization; WTO, World Trade Organization.

## Footnotes

1 Non-discrimination means that foreign investors are treated no differently than domestic investors who, it is useful to note, are not eligible to use ISDS, since these rules apply only to foreign investors.
